# Effects of Aerobic and Anaerobic Fatigue Exercises on Postural Control and Recovery Time in Female Soccer Players

**DOI:** 10.3390/ijerph17176273

**Published:** 2020-08-28

**Authors:** Özkan Güler, Dicle Aras, Fırat Akça, Antonino Bianco, Gioacchino Lavanco, Antonio Paoli, Fatma Neşe Şahin

**Affiliations:** 1 Faculty of Sports Sciences, Ankara University, Gölbaşı, Ankara 06830, Turkey; oguler@ankara.edu.tr (Ö.G.); daras@ankara.edu.tr (D.A.); fakca@ankara.edu.tr (F.A.); nesesahin@ankara.edu.tr (F.N.Ş.); 2 Department of Psychological, Pedagogical, Educational Science and Human Movement, University of Palermo, 90144 Palermo, Italy; gioacchino.lavanco@unipa.it; 3 Department of Biomedical Science, University of Padova, 35122 Padova, Italy; antonio.paoli@unipd.it

**Keywords:** balance, fatigue, female, support leg, recovery

## Abstract

Sixteen female soccer players (age = 20.19 ± 1.52 years; body mass = 56.52 ± 4.95 kg; body height = 164.81 ± 4.21 cm) with no history of lower extremity injury participated in the study. The Biodex SD Balance system was used to determine the non-dominant single-leg stability. In anaerobic exercise, each subject performed four maximal cycling efforts against a resistance equivalent to 0.075 kg/body mass for 30 s with three-minute rest intervals. In aerobic exercise, subjects performed the Bruce protocol on a motorized treadmill. After each exercise, subjects subsequently performed a single-leg stability test and then repeated the same test for four times with five-minute passive rest periods. In accordance with the results, it was found that the impairment observed right after the aerobic loading was higher (*p* < 0.001) compared to the anaerobic one. However, the time-related deterioration in both aerobic and anaerobic loadings was similar. The B-pre value was lower than B_post_ and B_5_ (*p* < 0.01) and B_10_ (*p* < 0.05) in both conditions. Subjects could reach the initial balance level at B_15_ after aerobic and anaerobic loadings. The lactate level did not reach resting value even after 20 min of both fatigue protocols. Although the fatigue after aerobic and aerobic exercise negatively affects a single-leg dynamic balance level, single leg balance ability returns to the baseline status after 10 min of passive recovery duration.

## 1. Introduction

The popularity of soccer among females is increasing each passing day. It is estimated that around 30 million females are actively playing licensed soccer in more than 100 countries around the world. Studies indicate that as the participation of females in soccer increases, the incidence of injury increases at a high rate [1,2,3]. In a soccer match, soccer players engage in many moves such as high-intensity acceleration, deceleration, sudden change of direction, bounce, and other soccer-oriented movements. Along with these moves, soccer players often experience various injuries when using one leg for stopping and cutting during pressure, while using the other leg to tackle the ball [4]. In addition to these, injuries in soccer are caused by sudden acceleration and deceleration without impact, rapid disorientation, and exposure to high loads while maintaining the stability of the knee joint in unpredictable movements [5,6,7,8]. When the injuries experienced in soccer were evaluated according to sex differences, It was reported that female athletes had a higher incidence of anterior cruciate ligament (ACL) experience in lower extremity injuries due to biomechanical and neuromuscular differences [5,6] than males [9].

Furthermore, previous studies indicate that female soccer player has the risk of ACL injury nine times greater than males [10]. Several risk factors cause these injuries in female soccer players. These risk factors in female soccer players include a previous history of injury [11], as well as a decline in hip strength [12] due to accumulated fatigue [11] and deterioration of lower extremity dynamic balance [13]. Epidemiological studies have pointed out that 50% of the injuries occur at the end of competitions or sports activities, and 58% of these injuries are due to non-impact conditions. That fatigue is an essential element of sensory-motor changes associated with injury [14,15]. Ekstrand et al. [9] report in their study that traumatic injuries occur more often in the last minutes of both halves of a soccer match [9]. In addition to these, it was reported in another study that lower extremity injuries were commonly seen at the last minutes of competition in sports such as soccer, which includes high-intensity moves and multi-directional sprints [9,16]. Therefore, it can be stated that non-contact injuries caused by fatigue occurred in the last fifteen minutes of play in both the first and second half of games. In non-contact injuries, neuromuscular fatigue is seen as a risk factor [17,18,19,20]. Neuromuscular fatigue is divided into two, according to the intensity and duration of exercise, like peripheral and central nervous system fatigue. Long-term activities affect the central nervous system, while short-term high-intensity activities cause peripheral fatigue [21,22]. Peripheral fatigue arises when there is not adequate energy provided to the muscles, despite the increasing energy need [22].

Moreover, it has been reported that muscle fatigue affects both peripheral and central proprioceptive processes [23,24,25]. Balance is defined as being able to hold the body center of gravity within the center of support [26]. In order to maintain balance visual, vestibular, and proprioceptive systems play a crucial role, and these systems are affected by many factors [27,28,29,30,31,32]. The proprioceptive system consists of the Golgi tendon organ, the muscle spindle, the Pacini corpuscle, free nerve endings, and the receptors in the joint capsules and skin [32,33,34]. It ensures maintenance of the balance with the information collected from these structures [32,34]. The proprioceptive system is affected by fatigue, aging, sarcopenia, neurological disease fibromyalgia, cancer, and rheumatological diseases and may result in impaired balance [35,36,37,38]. Many researchers have shown that fatigue negatively affects dynamic postural control [39,40,41,42]. Fatigue is an essential factor that acutely affects balance ability. In a study in which the center of pressure (COP) was measured before, in halftime and immediately after a soccer match, it was determined that the balance skill of the support leg was impaired in the post-match measurement [41]. The return of balance ability to average values after fatigue depends on many factors. The return of post-fatigue balance ability to initial level depends on the duration, intensity, and type of intensity of the fatigue protocol performed [43]. Deficits in dynamic postural control is a risk factor in experiencing falls and lower extremity injuries [13,42,44,45,46,47]. The deterioration of dynamic balance is associated with reactive and compensating movements, and it is stated that this impairment has been linked to falling risk and lower extremity injuries [18,48,49]. Since fatigue increases the rate of injury in athletes [9,16], and the lack of postural control is a lower extremity injury risk factor [13,50,51], it can be expected that the rate of injury as a result of fatigue-induced postural control (fatigue-induced balance deficits) may increase.

Soccer is classified as both an aerobic and an anaerobic sport. In the game, players may experience fatigue from time to time as a result of aerobic and anaerobic activities. There are studies in the literatüre on the effects of anaerobic fatigue on balance performance in soccer players. However, up to date, no previous studies have examined the effects of both types of fatigue in soccer players. Besides, many activities such as passing, kicking, and jumping in soccer are carried out on the support leg. This research will be the first to examine the effects of fatigue on support leg balance performance. Therefore, this study aims to determine the effects of different fatigue protocols on balance performance of the support-leg in female soccer players and to understand the time required for the balance to recover after loading.

## 2. Materials and Methods

Sixteen sub-elite female soccer players (with mean age of 20.19 ± 1.52 years, body mass 56.52 ± 4.95 kg, body height 164.81 ± 4.21 cm, percent body fat 22.63 ± 2.42%, and maxVO_2_ 52.33 ± 5.74 mL.kg.^−1^min^−1^) participated in the study voluntarily. Players who suffered lower extremity injuries for the last six months were not included in the study. Participants were instructed not to perform exercises that may cause exhaustion 48 h before the tests and not to use stimulants such as alcohol, caffeine, or drugs in the last 24 h before the study. The study was conducted according to the Declaration of Helsinki and was approved by the Ethics Committee of Ankara University, Approval code 21-1300-17, released in December 2017.

### 2.1. Balance Test

The participants were invited to participate in the Biodex SD Balance System (Biodex, Shirley, NY, USA) athletic single-leg testing protocol [52]. As noted, the Biodex Balance System (BBS) uses a circular platform that is free to move in the anterior-posterior and medial-lateral axes simultaneously. The BBS measures, in degrees, the tilt about each axis during dynamic conditions and calculates an overall stability index (OSI). A high score in the OSI indicates poor balance. The platform stability ranges from 1–12, with 1 representing the most significant instability.

The familiarization protocol was implemented before the experiments. All participants were instructed to perform the balance test on five different days of the targeted week.

**Figure 1:**
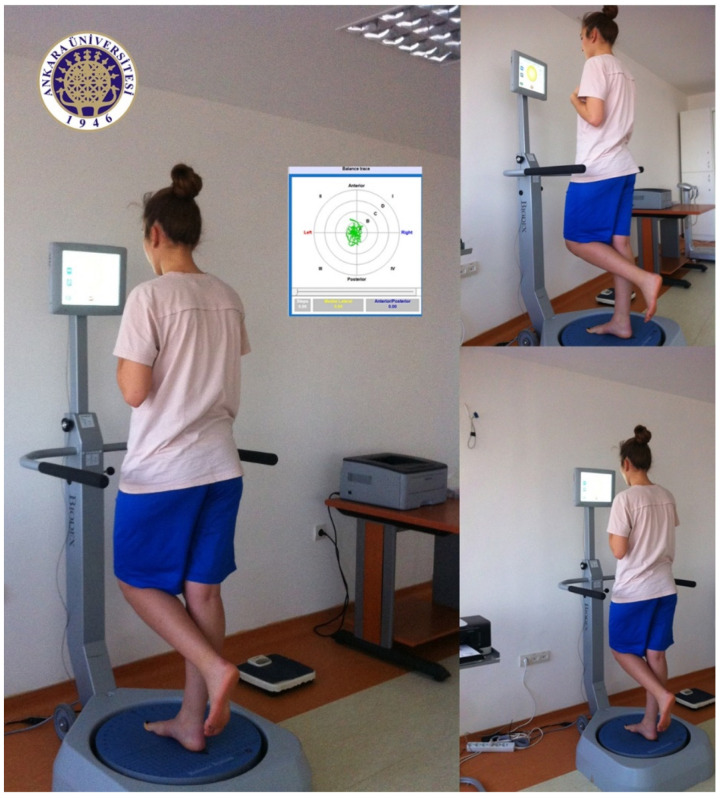
One subject during the data collection phase. Female soccer player.

The athletic single leg test protocol consisted of 3 trials of 20 s of upright stance on support-leg with 10 s of rest intervals between trials. Participants were asked to place their feet with the malleolar axis aligned with the midpoint of the platform over the center dot of the platform in a comfortable position. An athletic single-leg test was conducted on BBS with the platform set at level 4. Balance tests were carried out on the non-dominant leg. The same test protocol was performed before (B_pre_) and right after (B_post_) both aerobic and anaerobic fatigue protocols, and repeated at the 5th (B_5_), 10th (B_10_), 15th (B_15_), and 20th (B_20_) minutes. The participants were allowed to rest passively during the 5 min of recovery periods. There was a 2-day period between aerobic and anaerobic fatigue protocols (Figure 1).

### 2.2. Aerobic Fatigue Protocol

The Bruce protocol was performed on a motorized treadmill (Cosmed, Rome, Italy) in order to create aerobic fatigue in soccer players [53]. The participants continued the test until they were exhausted. At the end of the test, the maxVO_2_ consumption values of the participants were calculated and recorded. Rating of perceived exertion (RPE) was obtained using the 6–12 point Borg scale at the end of every load [54]. MaxVO_2_ was defined as the highest 30 s average in oxygen uptake and maximal heart rate (HRmax) as the highest every 10 s average during the Bruce protocol. A test was considered maximal when four of the following criteria were completed: VO2 plateau at peak exercise, respiratory exchange ratio ≥ 1.10 greater age-predicted maximal heart rate (220-age), and an indication of 18–20 rating on the Borg RPE scale [55].

### 2.3. Anaerobic Loading Protocol

Anaerobic fatigue protocol was performed using a bicycle ergometer (Monark Erogomedic 894 E Peak Bike Vansbro Sweden). The Wingate test protocol was used for anaerobic fatigue. In the Wingate protocol, participants were asked to pedal at maximal speed for 30 s. As the intensity of the training, a weight equivalent to 7.5% of the participants’ body mass was placed on the load scale. Once the participants started pedaling, the scale dropped when the bike’s wheel revolution went up to 150 rpm and the maximal pedaling for 30 s. Soccer players were verbally motivated during training. Participants repeated the Wingate test protocol a total of 6 times with intervals of 4 min.

### 2.4. Lactate Testing

Participants’ blood lactate values were determined immediately after the fatigue protocol and during the recovery period (L_pre_) just before the balance tests at 0th (L_post_), 5th (L_5_), 10th (L_10_), 15th (L_15_), and 20th (L_20_) minutes. During the lactate test, participants’ fingertips were wiped with alcohol-based tissue paper, and their capillary blood samples were taken with a lancet pen. Blood lactate levels of the participants were determined by an Accutrend Plus lactate device (Roche Diagnostics, Basel, Switzerland).

### 2.5. Statistical Analysis

In all statistical analyses, SPSS version 20 was used (SPSS Inc., Chicago, IL, USA). First, because the number of participants was below 50, the normality of the data was analyzed with the Shapiro-Wilk test. Depending on the distribution, lactate and balance values obtained at different times following aerobic and anaerobic fatigue protocols were compared by the Paired Sample t-Test or Wilcoxon Test. For the intergroup analyzes, either the Repeated Measurements Analysis of Variance (Aerobic Lpre, L5, L10, L15, L20; Bpre, B5, B15, B20; Anaerobic Lpost, L5, L10, L15, L20; Bpre, B5, B15, B20) or the Friedman test (Aerobic Lpost, Bpost, B10; Anaerobic Lpre, Bpost, B10) was used again depending on the distribution. In the case of the dataset exhibited both non-normally distributed and norma distributed data, we proceeded with the non-parametric analysis. In all statistical analyses, the alpha value was considered to be 0.05.

## 3. Results

The following variables were shown to be not normally distributed (Aerobic Lpost, Bpost, B10; Anaerobic Lpre, Bpost, B10).

The lactate values obtained from the participants before, during, and after aerobic and anaerobic fatigue protocols are shown in Table 1.

According to the results, there was no significant difference between participants’ resting lactate concentration values obtained before aerobic and anaerobic loading (*p* > 0.05). However, the lactate values obtained immediately after, 5th, 10th, 15th, and 20th min were statistically significant (*p* < 0.01) according to the fatigue conditions. After anaerobic loading, lactate values were found to be higher. Besides, it was understood that 20 min was not sufficient for the recovery, regardless of the type of loading.

Lactate values obtained at six different phases of the aerobic fatigue protocol were significantly different (*p* < 0.01). The resting lactate value was determined to be lower than all others reached after loading. Also, a significant difference was found between L_post_ and L_10_, L_15_ and L_20_; L_5_ and L_10_, L_post_ L_15_, and L_20_; L_10_ and L_15_ and L_20 (_*p* < 0.01); and with L_5_ and L_20_ (*p* < 0.05).

In lactate values measured after anaerobic loading, the resting value was determined to be significantly lower than in all other measurements. The difference between L_post_ and L_5_ was not significant, similar to the aerobic one. However, a significant difference at the level of *p* < 0.01 was found between L_post_ and L_10_, L_15_, and L_20_, between L_5_ and L_10_, L_15_, and L_20_ and between L_15_ and L_20_.

**Figure 2:**
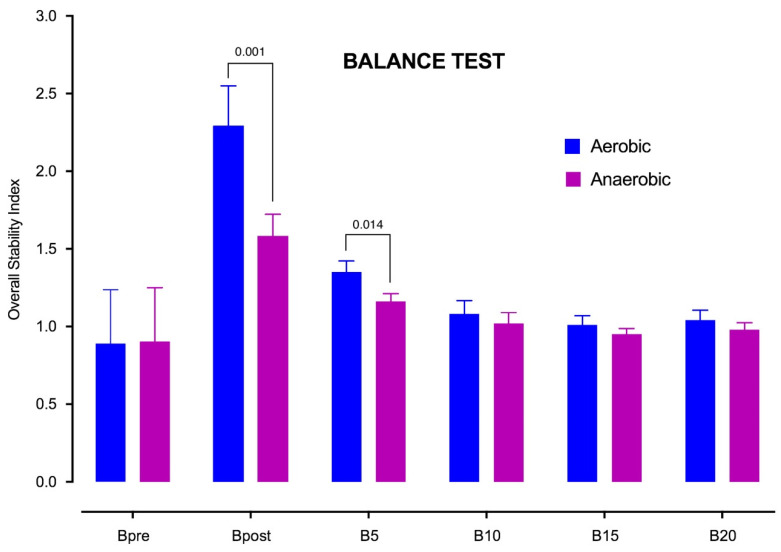
Overall stability index across the experimentation. Aerobic condition vs. anaerobic condition.

When the results related to the balance were examined, a significant difference was found at the level of *p* < 0.01 between the balance values obtained immediately after aerobic and anaerobic loading and at the 5th minute. It was understood that there is more deterioration in the balance after aerobic loading. No significant difference was observed in the values obtained after 10th, 15th, and 20th min, depending on the type of loading (Figure 2).

When the values obtained due to aerobic loading are taken into account, it was understood that the differences between B_pre_ and B_post_, B_5_ (*p* < 0.01), and between B_pre_ and B_10_ were (*p* < 0.05) statistically significant. However, there was no significant difference with the values reached between B_15_, B_20_, and B_pre_. Accordingly, it can be stated that recovery takes place after aerobic load in the state of balance between 10th and 15th minutes. In the other results, on the other hand, a significant difference was found between B_post_ and B_5_, B_10_, B_15_, and B_20_ (*p* < 0.01). The measurement of B_5_ was found to be significantly higher than the measurements of B_10_, B_15_ and B_20_ (*p* < 0.01).

## 4. Discussion

This study aimed to investigate the acute effects of aerobic and anaerobic exercises on dynamic balance skill and recovery time in female soccer players. In order to control the fatigue level, lactate concentrations of the subjects were also collected. According to the lactate test results, both fatigue protocols were found to be successful in creating fatigue. Although it was higher after the anaerobic exercise, the lactate level did not return to the initial level within 20 min after both fatigue conditions. Besides, as a result of strenuous aerobic or anaerobic exercise, female soccer players’ ability to balance the support leg was affected negatively. After both aerobic and anaerobic loading, the recovery time of balance skill lasted about 10 min. The deterioration in the athletic single-leg stability test was observed to be higher after the aerobic fatigue protocols in all of the measurements. Furthermore, the difference was statically significant in B_post_ (*p* < 0.01) and B_5_ (*p* < 0.05) values. In the literature, many studies suggest that aerobic and anaerobic fatigue negatively affect balance ability [41,42,43,56,57,58,59,60,61]. There are similar studies on this subject in the literature. In a study in which both aerobic and anaerobic fatigue protocol was implemented, and the balance level was determined with the Balance Error Scoring System (BESS), no difference was found between the balance performance and its time of recovery after both fatigue protocols. However, athletes returned to their initial balance performance values within 8–13 min after both fatigue protocols [58]. Steinberg et al. investigated the balance level after a Yo-Yo test with the Interactive Balance System (Tetrax) device and reported that balance skill returned to the initial level within 10 min after fatigue [62]. In the current study, balance skills returned to the initial level after approximately 10–15 min after both aerobic and anaerobic fatigue. Moreover, the present study found no difference between balance levels during the recovery time after aerobic or anaerobic fatigue. In a study conducted on a bicycle ergometer, participants performed two maximal Wingate tests lasting 30 s with a rest interval of 2 min. At the end of high-intensity activation, it was determined that balance skill was affected negatively and returned to the baseline level within 10 min [63]. Ishizuka et al. applied the functional fatigue protocol to 14 male and 9 female college-level soccer players and determined the balance with the Biodex Limit of Stability Test. As a result, the subjects were found to have returned their initial level within 10 min after the fatigue [59]. In a study by Matsuda et al., a functional fatigue protocol was applied to 100 recreationally active college students. After the functional fatigue protocol, it was reported in the measurements made with the balance error score system that the balance performance returned to its initial level within approximately 20 min [64]. Although a similar fatigue protocol was implemented, different balance performance recovery times were observed between the study mentioned above and our study. This difference is thought to be since soccer players have a better balance skill than other athletes and sedentary people. Contrary to the result of the current study, it is reported in some studies that the recovery time of balance ability lasts longer than 10 min, while in some studies, it is reported that balance ability is not affected after fatigue. In a study where the fatigue protocol involving sports activities was applied, it was reported that the participants’ balance values returned to their initial values after 20 min as a result of the balance test conducted with the balance error score system [56]. In another study, in which balance measurements were made after a 25-min treadmill run, it took approximately 15 min for the athletes participating in the study to return their balance performance to its initial level [65]. In another study, where balance measurement was performed with the biodex balance system before and during a soccer match, it was determined that the dominant leg balance performance of the players was impaired while no change was observed in the support leg balance performance [66]. In contrast to this, soccer players’ support leg balance skills were found to be negatively affected following the fatigue protocol in the present study. The reason for the difference is thought to be due to the degree of difficulty differences between the protocols of balance tests. In the literature, some studies did not observe any changes experienced in balance performance after fatigue. After soccer-specific fatigue [67] and after soccer training [68], balance measurements made with the Biodex Balance System have reported that the balance performance of soccer players is not affected by fatigue. Paillard reported that the return of post-fatigue balance ability to initial values depends on the duration, density, and intensity of the fatigue protocol performed [43]. In these studies, it is thought that the reason why there is no difference after fatigue is due to insufficient density and intensity of the fatigue protocol for impaired balance ability. In addition, longer times to return to initial values of balance performance were reported in these studies compared to the current study. The reason for these varied results may have been the difference between the branches of the athletes participating in the studies or the difference in balance measurement methods.

## 5. Conclusions

As a result of this study, it was clearly observed that balance performance is impaired in soccer players after both aerobic and anaerobic fatigue. The impairment of fatigue and balance performance are seen as significant risk factors. Although there is not enough data on the effects of fatigue on balance ability in soccer players, it is stated in several studies that fatigue increases the incidence of injury experienced [69] and that deterioration in balance performance may increase ankle injuries [70]. Many researchers also suggest that balance training should be performed to prevent injuries [1,71,72]. Therefore, trainers should give importance to balance training in order to prevent non-contact injuries caused by loss of balance. In future studies, it is suggested to investigate the effects of fatigue on the balance ability in athletes performing balance training.

The study was performed exclusively on healthy young adult female soccer players (who suffer from a higher prevalence of non-contact ACL injuries). A limitation of the study could be seen as a lack of control of the menstrual cycle. In addition, in this study, anaerobic fatigue protocol was performed with a bicycle ergometer and aerobic fatigue with a treadmill. The measurement of balance performance after a real soccer match is thought to provide a clearer picture of the effects of soccer-specific fatigue mechanisms on balance performance. 

## Figures and Tables

**Table 1:** Mean comparisons of participants with lactate and balance, which vary depending on aerobic and anaerobic loading. In horizontal, the repeated measures *p* values, in vertical the *p* values for aerobic vs. anaerobic comparisons.

**Fatigue Protocol**	**L_pre_**	**L_post_**	**L_5_**	**L_10_**	**L_15_**	**L_20_**	***p_***
Aerobic	1.20 ± 0.36	11.70 ± 2.53	11.81 ± 2.51	9.86 ± 2.58	7.84 ± 2.15	6.93 ± 1.87	0.000 **
Anaerobic	1.18 ± 0.33	15.43 ± 2.40	15.09 ± 2.27	13.76 ± 2.50	11.25 ± 2.14	9.49 ± 2.51	0.000 **
***p_***	0.823	0.001 **	0.000 **	0.000 **	0.000 **	0.001 **	-
**Fatigue Protocol**	**B_pre_**	**B_post_**	**B_5_**	**B_10_**	**B_15_**	**B_20_**	***p_***
Aerobic	0.89 ± 1.39	2.29 ± 1.04	1.35 ± 0.29	1.08 ± 0.35	1.01 ± 0.24	1.04 ± 0.26	0.000 **
Anaerobic	0.90 ± 1.40	1.58 ± 0.57	1.16 ± 0.21	1.02 ± 0.28	0.95 ± 0.15	0.98 ± 0.18	0.000 **
***p_***	0.745	0.001 **	0.014 *	0.308	0.410	0.509	-

L: Lactate; B: Balance; * *p* < 0.05; ** *p* < 0.01.
